# Asialoglycoprotein receptor targeted delivery of doxorubicin nanoparticles for hepatocellular carcinoma

**DOI:** 10.1080/10717544.2016.1225856

**Published:** 2017-02-03

**Authors:** Sandhya Pranatharthiharan, Mitesh D. Patel, Vinod C. Malshe, Vaishali Pujari, Ajit Gorakshakar, Manisha Madkaikar, Kanjaksha Ghosh, Padma V. Devarajan

**Affiliations:** 1 Department of Pharmaceutical Sciences and Technology, Institute of Chemical Technology (Deemed University, Elite Status), Mumbai, Maharashtra, India and; 2 National Institute of Immunohaematology, Mumbai, Maharashtra, India

**Keywords:** Pullulan, arabinogalactan, doxorubicin hydrochloride, asialoglycoprotein receptor, PLC/PRF/5 liver tumor, hepatocellular carcinoma

## Abstract

We report asialoglycoprotein receptor (ASGPR)-targeted doxorubicin hydrochloride (Dox) nanoparticles (NPs) for hepatocellular carcinoma (HCC). Polyethylene sebacate (PES)-Gantrez® AN 119 Dox NPs of average size 220 nm with PDI < 0.62 and ∼20% Dox loading were prepared by modified nanoprecipitation. ASGPR ligands, pullulan (Pul), arabinogalactan (AGn), and the combination (Pul-AGn), were anchored by adsorption. Ligand anchoring enabled high liver uptake with a remarkable hepatocyte:nonparenchymal cell ratio of 85:15. Furthermore, Pul-AGn NPs exhibited an additive effect implying incredibly high hepatocyte accumulation. Galactose-mediated competitive inhibition confirmed ASGPR-mediated uptake of ligand-anchored NPs in HepG2 cell lines. Subacute toxicity in rats confirmed the safety of the NP groups. However, histopathological evaluation suggested mild renal toxicity of AGn. Pul NPs revealed sustained reduction in tumor volume in PLC/PRF/5 liver tumor-bearing Nod/Scid mice up to 46 days. Extensive tumor necrosis, reduced collagen content, reduction in the HCC biomarker serum α-fetoprotein (*p* < 0.05), a mitotic index of 1.135 (day 46), and tumor treated/tumor control (T/C) values of <0.42 signified superior efficacy of Pul NPs. Furthermore, weight gain in the NP groups, and no histopathological alterations indicated that they were well tolerated by the mice. The high efficacy coupled with greater safety portrayed Pul Dox NPs as a promising nanocarrier for improved therapy of HCC.

## Introduction

Hepatocellular carcinoma (HCC) is the third most prevalent and deadliest cancer with a mean survival time of barely six months (El-Serag & Rudolph, 2007). It primarily afflicts hepatocytes which represent more than 80% of the hepatic cells (Poelstra et al., 2012). However, rapid sequestration of particulate carriers by kupffer cells severely limits the hepatocyte accumulation, seriously compromising therapeutic efficacy. Targeted delivery to hepatocytes therefore construes an important strategy for HCC therapy and can be achieved by targeted delivery to receptors that are overexpressed on hepatocytes (Han et al., 1999).

Delivery of Dox nanoparticles to hepatocytes through glycyrrhetinic acid receptor is demonstrated (Zhang et al., 2012; Mezghrani et al., 2015). Paclitaxel liposomes (Chen et al., 2015) and Dox nanoparticles (Zhang et al., 2016) were targeted to hepatocytes through the integrin receptor. Although other receptors on hepatocytes such as the retinoic acid receptor (Varshosaz et al., 2013), bile receptor (Pütz et al., 2005), hyaluronan receptor (Kumar et al., 2015), low-density lipoprotein (LDL), and high-density lipoprotein (HDL) receptor (Jain et al., 2013) may be targeted, the asialoglycoprotein receptor (ASGPR), a hepatic lectin, provides an ideal target for hepatocyte-specific delivery. It is expressed in abundance on hepatocytes (500 000 ASGPR/hepatocyte) and minimally present elsewhere in the body. Other benefits include ease of access from the vascular compartment and the ability to internalize large molecules through clathrin-mediated endocytosis (McAbee et al., 2000; D’Souza & Devarajan, 2015a,b). Moreover, the affinity of the ASGPR for simple ligands like carbohydrates is a major advantage (Benjamin & Robinson, 2002; Li et al., 2008). Lactose-anchored polyethylene glycol (PEG) Dox nanoparticles exhibited superior inhibition against hepatoma cells (Lu et al., 2011). While galactose branched-cyclodextrins enabled hepatocyte targeting *in vitro* (Shinoda et al., 1998), paclitaxel-loaded galactose-installed micelles exhibited high efficacy with reduced toxicity in human hepatoma SMMC-7721 tumor-bearing nude mice (Zou et al., 2014). ASGPR-mediated targeting with promise in HCC was also demonstrated using Dox-loaded galactosamine-conjugated albumin nanoparticles (Shen et al., 2011) and lactoferrin-conjugated PEGylated liposomes (Wei et al., 2012).

The high affinity of arabinogalactan (a galactose-based polymer) and pullulan (a glucose-based polymer) to ASGPR has been confirmed in silico through molecular docking (Kaneo et al., 2000; D’Souza et al., 2013). Arabinogalactan (AGn), consisting of arabinose and galactose monosaccharides, is a polysaccharide from the plant *Larix occidentalis*. It is rapidly removed from the blood by the liver after intravenous injection making it a viable liver targeting carrier (Kaneo et al., 2000; Tanaka et al., 2004). Suppressed serum viral DNA titers in woodchucks infected with hepatitis virus, a hepatocyte affliction, suggested hepatocyte targeted delivery of larch arabinogalactan conjugated with the antiviral agent vidarabine monophosphate (Prescott et al., 1995). Dox liposomes with palmitoylated arabinogalactan as a targeting ligand exhibited promise in HCC (Shah et al., 2014). Moreover, the anti-metastatic properties of AGn could provide an added advantage (Beuth et al., 1987).

Pullulan, a linear polysaccharide with maltotriosyl repeating units, is a nontoxic, nonimmunogenic, nonmutagenic, and noncarcinogenic polysaccharide (Shingel, 2004). A polyethyleneimine–pullulan conjugate was successfully used for liver cell gene delivery (Rekha & Sharma, 2011). A pH-sensitive pullulan-based nanoparticle carrier of methotrexate and combretastatin A4 was successfully developed as a combination therapy for HCC (Wang et al., 2013), whereas a pullulan-based polymeric prodrug of cisplatin demonstrated superior efficacy in MHCC-97H liver tumor-bearing nude mice (Wang et al., 2015). Furthermore, prolonged *in vivo* blood circulation time of Pul is reported (Wang et al., 2015).

We have earlier reported circulation longevity of pullulan functionalized polyethylene sebacate (PES)-Gantrez® AN 119 Dox nanoparticles with the possibility of lower cardiotoxicity (Guhagarkar et al., 2009,2010). As Pul and AGn are known ASGPR ligands (Prescott et al., 1995; Rekha & Sharma, 2011), in the present study, we evaluate PES-Gantrez® AN 119 Dox NPs anchored with Pul, AGn, and a combination of Pul with AGn (Pul-AGn) as ligands for ASGPR-mediated hepatocyte targeted delivery *in vitro* and anticancer efficacy in PLC/PRF/5 liver tumor-bearing Nod/Scid mice as a preclinical xenograft model of HCC.

## Materials and methods

### Materials

Doxorubicin hydrochloride (Dox) was obtained from Khandelwal Laboratories, Mumbai as a gift sample. PES was synthesized in our laboratory (molecular weight: 9000–11000). Gantrez® AN 119 (poly(methyl vinyl ether-co-maleic anhydride) with an average molecular weight of 200 kDa was obtained as a gift sample from Ashland India. Tween 80, glacial acetic acid AR, tetrahydrofuran AR (THF), and acetone AR were purchased from s.d. fine Chemicals (Mumbai, India). Magnesium acetate tetrahydrate pure and sodium acetate trihydrate crystal pure were purchased from MERCK (Mumbai, India). Acetonitrile HPLC grade was supplied as a gift sample by Azeocryst Organics Pvt. Ltd., Mumbai, India. Trehalose and pullulan (repeated glucose units; α-1,4;1,6-glucan, molecular mass of 200 kDa, Hayashibara, Japan) were supplied as gift samples by Gangwal Chemicals Pvt Ltd (Mumbai, India). Arabinogalactan (galactopyranose:arabinofuranose 6:1; 3,6-β-D-galactan with β-galactose and β-arabinose side chains; average molecular weight: ∼19 kDa) was purchased from Sigma, Mumbai. Collagenase type I was purchased from Sigma Aldrich. D (+) galactose (molecular weight: 180.16) was purchased from s.d. fine chemicals Ltd. Filtered (0.45-μ membrane filter) doubled distilled water was used for preparation of nanoparticles. All the other chemicals and reagents were either of spectroscopic or of analytical grade. Serum biochemistry and histopathology of rat organs were carried out at Unique Bio Diagnostic Enterprises, Mumbai. Histopathology of tumor, kidney, and heart of tumor-bearing mice was carried out at Animal Health Care Center, Mumbai. Evaluation of serum α-fetoprotein was carried out at Unique Bio Diagnostic Enterprises, Mumbai.

### Preparation and characterization of PES-Gantrez**®** an 119 Dox nanoparticles

PES-Gantrez® AN 119 Dox nanoparticles were prepared by modified nanoprecipitation technique as reported earlier (Guhagarkar et al., 2009,2010). Briefly, the polymers were dissolved in THF and acetone (1:1) as the organic phase (10 ml). Aqueous phase (25 ml) consisted of Dox (10 mg) and tween 80 (10% v/v). Drop-wise addition of organic phase to the aqueous phase under magnetic stirring was followed by the addition of aqueous magnesium acetate tetrahydrate (1 ml of 0.5% w/v). The resulting nanoparticle dispersion was stirred for a period of 4 h for complete evaporation of the organic phase and then probe sonicated (DP120, Dakshin Mumbai, India) for a period of 10 min with 15 sec on/off cycle. Isolation of nanoparticles was done by centrifugation (14 000 rpm for 40 min), and the pellet was dispersed in purified water. Pullulan (Pul), arabinogalactan (AGn), and Pul-AGn combination with nanoparticles:ligand ratio of 1:1 were added to the nanoparticle dispersion, cyclomixed, and probe sonicated (10 min, 15 sec on/off cycle) and allowed to stand for 10 min. To determine the entrapment efficiency, nanoparticles were separated from the dispersion by centrifugation at 14 000 rpm for 40 min. The supernatant obtained after centrifugation was suitably diluted and analyzed for free Dox by UV spectrophotometry (Shimadzu, Japan) at 478 nm. The % EE was calculated as follows

(1) $$\%\;\mathrm{EE}\;=\;\left({\left[\mathrm{Dox}\right]}_{\mathrm{total}}\;-\;{\left[\mathrm{Dox}\right]}_{\mathrm{supernatant}}\right)/{\left[\mathrm{Dox}\right]}_{\mathrm{total}}\times 100$$

Dox loading was calculated based on the weight ratio of the loaded amount of Dox to the amount of nanoparticles.

Particle size was determined by photon correlation spectroscopy using N4 plus submicron particle size analyzer (Beckman Coulter) at 25 °C with all the measurements taken by scattering the light at 90°. Zeta potential of nanoparticles was determined using Zeta sizer Nano ZS (Malvern Instruments Ltd, Malvern UK).

### Biodistribution study

Female Sprague Dawley rats (200–250 gms) fasted for 12 h were divided into five groups (*n* = 6): Group I, Dox solution; Group II, NPs; Group III, Pul NPs; Group IV, AGn NPs; and Group V, Pul-AGn NPs. The formulations with Dox equivalent to 6 mg/kg body weight were administered intravenously through the tail vein. Rats were euthanized at 1 h, 6 h, and 24 h and the brain, liver, lungs, spleen, kidneys, and heart were isolated. The organs were placed in phosphate-buffered saline (PBS), pH 7.4, and homogenized using a tissue homogenizer. To 500 μl of the homogenate, 500 μl of acetonitrile was added and the sample vortex mixed for 5 min, followed by sonication for 5 min, and centrifugation at 20 000 rpm for 20 min. The supernatant was injected into the HPLC system (Jasco LC 1500) coupled with UV detector (Jasco UV/VIS 1570/1575) and Rheodyne injector model fitted with 100 μl sample loop. Chromatography was performed on a Waters Spherisorb® S5 ODS2 (250 × 4.6 mm i.d., 5 μm particle size) column and a C18 guard column at room temperature under isocratic conditions at a flow rate of 1 ml/min with UV detection at a *λ*_max_ of 254 nm. The mobile phase comprised of acetonitrile:methanol:acetate buffer pH 5.0 (25:30:45 v/v), whereas for brain homogenate, the mobile phase comprised of acetonitrile:methanol:acetate buffer pH 5.0 (20:30:50 v/v). Dox was analyzed by HPLC and compared with calibration graph against appropriate blank.

### Intrahepatic disposition

The intrahepatic disposition of Dox formulations was evaluated in female Sprague Dawley rats (200–250 gms, *n* = 4). Rats were divided into five groups: Group I, Dox solution; Group II, NPs; Group III, Pul NPs; Group IV, AGn NPs; and Group V, Pul-AGn NPs. The formulations with Dox equivalent to 6 mg/kg body weight were administered to the rats by tail vein. At the end of 1 and 24 h, a combination of ketamine (70 mg/kg) and xylazine (5 mg/kg) was used to anesthetize the rats by intraperitoneal injection (0.2 ml). Abdominal cavity was opened, and the portal vein was cannulated with an angiocatheter connected to a perfusion pump. Perfusion was initiated at a rate of 4 ml/min with a Ca^2+^-^ ^and Mg^2+^-free perfusion buffer comprising 10 mM N-2-hydroxyethylpiperazine-N-2-ethanesulfonic acid (HEPES), 137 mM NaCl, 5 mM KCl, 0.5 mM NaH_2_PO_4_, and 0.4 mM Na_2_HPO_4_, pH 7.2, and continued up to 10 min. Then, the vena cava and aorta were cut, and perfusion continued by the same perfusion buffer supplemented with 5 mM CaCl_2_ and 0.05% (w/v) collagenase (type I, pH 7.5) for further 10 min. At the end of the perfusion, the liver was excised, its capsular membranes were removed, and the cells were dispersed in ice-cold Hank’s HEPES buffer by gentle stirring. This was followed by filtration through a 100-μm nylon cloth. The dispersed cells were separated by centrifugation at 5000 rpm for 1 min. The pellets containing hepatocytes were washed twice with Hank’s HEPES buffer by centrifuging at 500 rpm for 1 min. The supernatants of the washings which contained the nonparenchymal cells (NPC) were similarly centrifuged at least twice at 2000 rpm for 2 min. The hepatocytes and NPC were resuspended separately in Hank’s HEPES buffer (Joshi & Devarajan, 2014) and 0.1 ml transferred to a 1-ml Eppendorf tube. The cells were deproteinized with acetonitrile, and the mixture was vortexed and centrifuged; Dox was analyzed by HPLC as described earlier using Aquasil-C18 column with the mobile phase of acetonitrile:methanol:acetate buffer pH 5.0 (25:25:50 v/v).

### *In vitro* ASGPR-mediated uptake in HepG2 cell lines

ASGPR-mediated cellular uptake of Dox-loaded nanoparticles was evaluated in the human hepatocellular carcinoma cell line HepG2, known to overexpress the asialoglycoprotein receptors.

HepG2 cells were seeded in 96-well plates at densities of 2 × 10^5^ cells/well/0.2 ml of medium and allowed to adhere by incubating for a period of 24 h at 37 °C. The medium was discarded and incubated with Dox solution/Dox NPs 200 μl equivalent to 50 μg/ml of Dox at 37 °C for 1 h. At the end of 1 h, supernatant was collected and the cells in the wells were lysed using 0.5% w/v sodium lauryl sulfate solution (200 μL). The Dox content in the cell lysate was determined by HPLC. HepG2 cells were treated with aqueous galactose 65 mM for 1 h, and incubated with Dox solution/Dox NPs 200 μl equivalent to 50 μg/ml of Dox at 37 °C for 1 h (Huang et al., 2008). The cells were deproteinized with acetonitrile, and the mixture was vortexed and centrifuged; Dox was analyzed by HPLC as described earlier using Aquasil-C18 column with the mobile phase of acetonitrile:methanol:acetate buffer pH 5.0 (25:25:50 v/v).

### Toxicity study

Toxicity study was carried out in female Sprague Dawley rats (180–220 g, *n* = 5). Rats were divided into six groups: Group I, untreated control; Group II, Dox solution; Group III, NPs; Group IV, Pul NPs; Group V, AGn NPs; and Group VI, Pul-AGn NPs. All the animals of treatment groups received two doses of 6 mg/kg on day 1 and day 15. Weight of all the animals was recorded prior to treatment. At the end of day 28, all the animals were anesthetized (using combination of halothane and oxygen) and blood samples were collected from retro-orbital plexus region with a glass capillary. The rats were sacrificed by excessive carbon dioxide and organs including heart, liver, lungs, spleen, kidneys, and brain were isolated and examined for histopathology. Tissues and serum were preserved below −20 °C till complete analysis was performed. Weight of rats, and the heart and kidneys of rats of various treatment groups were monitored on day 28. Serum was analyzed for renal markers (serum creatinine, blood urea nitrogen, serum total protein, and serum total albumin), liver markers (serum glutamic pyruvate transaminase, serum glutamic oxaloacetic transaminase, and alkaline phosphatase), and cardiac markers (serum creatine kinase, troponin-I levels) using standard assay kits.

### *In vivo* efficacy in PLC/PRF/5 liver tumor-bearing Nod/Scid mice

*In vivo* testing for anticancer activity evaluation of Dox solution and Dox nanoparticles in the liver xenograft mouse model was done at Anti-Cancer Drug screening facility (ACDSF) at ACTREC, Tata Memorial Center, Navi Mumbai. The experiments were performed according to protocols approved by IAEC, ACTREC, Tata Memorial Center, Navi Mumbai and were covered by a CPSEA license for these studies. PLC/PRF/5 liver tumor models were established subcutaneously. Briefly, PLC/PRF/5 cells (1 × 10^7^/0.2 ml) were injected subcutaneously into the back of mice to establish a model of tumor-bearing mice. Approximately, 45-day post-implantation, the tumors were extracted. After necrotic tissue and noncancerous tissue were removed, the remaining cancerous tissue was cut into small pieces approximately 2 × 2 mm in size and transplanted to Nod/Scid mice. When tumor diameter reached ≥5 mm, the animals were divided into four groups (*n* = 6). Group I was the control group without any treatment, Group II, Group III, and Group IV received Dox solution, Pul NPs, and Pul-AGn NPs, respectively, in 5% dextrose (0.2 ml). The mice were treated on days 1, 4, and 7 by intravenous (i.v.) injections through tail vein of either Dox solution (2.5 mg/kg) or NPs (equivalent to Dox 2.5 mg/kg) (Akao et al., 2010; Khandekar et al., 2014; Derajram & Devarajan 2015). The animals were maintained under standard laboratory conditions (14 h:10 h dark/light cycle and a temperature of 22 ± 2 °C). The body weights of the mice were measured thrice a week. Antitumor activity was evaluated by monitoring the tumor growth rate and the tumor size was measured using slide calipers.

The tumor volume was measured. The tumor size was determined with a caliper in two dimensions and calculated using the following equation:

(2) $$V\;=\;\text{the longest diameter of tumor}\;*\;\;{\left(\text{the shortest diameter of tumor}\right)}^{2}/2$$

At the end of 32 and 46 days, mice were sacrificed by cervical decapitation, organs such as heart, kidneys, tumor were removed and subjected to histopathology studies. Collagen content of tumor was visualized by Masson’s trichrome staining and serum α-fetoprotein was evaluated as the liver tumor marker.

### Statistical analysis

All the values are expressed as mean value ± SD of at least three independent experiments. Statistical analysis was performed using the one-way ANOVA with Dunnet’s test, Bonferroni t test, and Student’s t-tests. *p* < 0.05 was the criterion for statistical significance.

All the experimental procedures were reviewed and approved by the Institutional Animal Ethics Committee (IAEC) of Institute of Chemical Technology, Department of Pharmaceutical Sciences and Technology, Mumbai, India (ICT/IAEC/2011/P72, ICT/IAEC/2012/P51, and ICT/IAEC/2012/P21).

## Results and discussion

### Physicochemical characterization of PES-Gantrez**®** AN 119 Dox-loaded nanoparticles

PES-Gantrez® AN 119 Dox NPs were successfully prepared by modified nanoprecipitation. High entrapment of  >70%, average size of 220 nm, zeta potential of −27 mv, and drug loading of around 20% w/w were correlated well with our previous report (Guhagarkar et al., 2010). An increase in size by ≈22–28 nm coupled with increase in zeta potential by ≈7 mv confirmed the anchoring of the ligands. The nanoparticles exhibited a biphasic drug release pattern with an initial burst release, followed by sustained release ascribed to the polymeric matrix imparting a barrier to drug release. Dox release was faster at pH 5.5 (t50% = 15 h) compared to pH 7.4 (Pranatharthiharan et al., 2016).

### Biodistribution study

**Figure 1.**
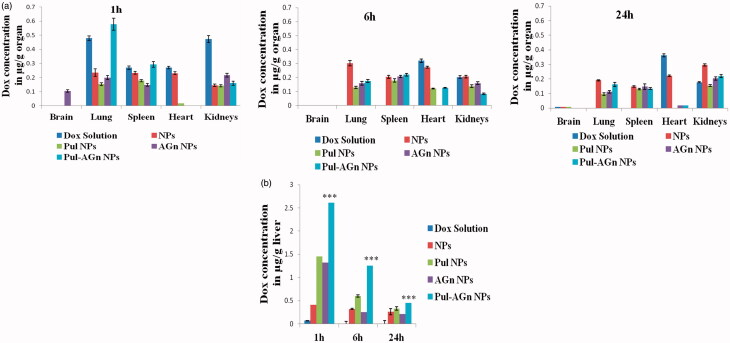
(a) Biodistribution profile of Dox formulations (b) Dox uptake in liver from Dox formulations, ***p < 0.001.

A significantly altered biodistribution profile was exhibited by Dox nanoparticles compared to Dox solution. The ligand-anchored nanoparticles exhibited significantly lower levels in the heart compared to Dox solution (*p* < 0.05, Figure 1a), indicating a major benefit of decreased cardiotoxicity, thereby overcoming one serious side effect of Dox solution. Levels in the kidney although significantly lower at 1 h (*p* < 0.01) were comparable with Dox solution at later time points. Nevertheless, targeted Dox delivery combined with diminished cardiac concentrations can minimize the impact of kidney toxicity (Manil et al., 1994,1995). Minimal brain uptake of Dox was evident only with AGn NPs and only at 1 h. Nanoparticles exhibited significantly higher liver uptake compared to Dox solution. At 1 h, while nanoparticles without ligands (NPs) exhibited a 6-fold enhancement in total liver uptake, Pul NPs and AGn NPs exhibited an almost 20-fold enhancement (Figure 1b). A striking finding was the 38-fold enhancement in liver uptake seen with Pul-AGn NPs predominant at all time points up to 24 h (*p* < 0.01) which implied an additive effect with the ligand combination. Furthermore, a sustained decrease in concentration was seen with Pul NPs and Pul-AGn NPs over 24 h. The rapid fall in concentration (*p* < 0.05) exhibited by AGn NPs is attributed to the lower molecular weight of AGn (19 kDa), resulting in faster elimination. The molecular weight of Pul was significantly higher (200 kDa) (D’Souza & Devarajan, 2016).

### Intrahepatic disposition

High liver uptake at 1 h and 24 h (*p* < 0.01) with a high hepatocyte:nonparenchymal cell (H:NPC) ratio of  >85:15 revealed by the ligand-anchored nanoparticles (Table 1) confirmed the extensive hepatocyte accumulation. In contrast, NPs revealed significantly low liver uptake although a high H:NPC ratio of 70:30 was observed. This high ratio is attributed to stealth enabled kupffer cell bypass due to the hydrophilicity and negative surface charge imparted by Gantrez® AN 119 (D’Souza & Devarajan, 2015a). On the other hand, Dox solution exhibited low liver uptake and was barely detectable in the hepatocytes. A minimum 70-fold enhancement in hepatocyte concentration was seen with the ligands compared to Dox solution, whereas Pul-AGn combination exhibited a near 2-fold enhancement compared to the individual ligands suggesting an additive effect (*p* < 0.05).

**Table 1.** Intrahepatic disposition of Dox formulations (mean ± SD; *n* = 4).

Formulations	Hepatocytes(H) (μg)	NPC(μg)	H:NPC ratio	Fold enhancement in hepatocyte accumulationReference: Dox solution
1 h				
Pul-AGn NPs	13.99 ± 0.24	1.78 ± 0.12	88.7:11.3	134.51
AGn NPs	7.08 ± 0.49	1.02 ± 0.10	87.4:12.6	68.07
Pul NPs	7.95 ± 0.16	1.16 ± 0.06	87.3:12.7	76.44
NPs	1.78 ± 0.17	0.814 ± 0.12	68.6:31.4	17.11
Dox solution	0.10 ± 0.02	0.154 ± 0.04	40.3:59.7	Reference
24 h				
Pul-AGn NPs	2.38 ± 0.13	0.38 ± 0.06	86.2:13.8	
AGn NPs	1.11 ± 0.10	0.18 ± 0.03	86:13.9	
Pul NPs	1.71 ± 0.07	0.28 ± 0.03	85.9:14	
NPs	1.09 ± 0.10	0.45 ± 0.08	70.8:29.2	
Dox solution	Not detected	Not detected		Reference

Although both Pul and AGn are reported to exhibit high affinity to ASGPR, some differences in their interaction with the receptor are identified (D’Souza et al., 2013). Although AGn revealed a strong interaction with Ca, Pul revealed binding to the amino acids Asp 259, Arg 236, and Glu 238, which is not seen with AGn. The additive enhancement in hepatocyte uptake exhibited by the ligand combination is ascribed to these differences.

### *In vitro* ASGPR-mediated uptake in HepG2 cell line

Competitive galactose uptake study (Huang et al., 2008) was carried out to confirm the role and specificity of the ASGPR to internalize the ligand-anchored NPs. The HepG2 cell line, a hepatocellular carcinoma cell line known to overexpress ASGPR (Zhang et al., 2014), was relied on for this study.

Ligand-anchored nanoparticles in the absence of galactose exhibited significantly higher uptake (*p* < 0.001) than NPs (22.79% ± 0.27) and Dox solution (12.35% ± 0.04) with Pul-AGn NPs (85.35% ± 0.33) exhibiting an additive effect compared to individual ligands (Pul NPs: 43.84% ± 0.30 and AGn NPs: 53.57% ± 0.27). Dox uptake was significantly suppressed from the ligand-anchored nanoparticles (Pul NPs: 11.62% ± 0.12, AGn NPs: 17.78% ± 0.11, Pul-AGn NPs: 22.60% ± 0.16; *p* < 0.001) in the presence of galactose after 1 h of incubation. This observed reduction in the uptake is due to the binding and internalization of the ASGPR follow Michaelis–Menten kinetics as explained in an earlier study (Schwartzs et al., 1982). Due to the high affinity of galactose for ASGPR, excess free galactose can effectively compete for and saturate the ASGPR and reduce their availability to bind and internalize the ligand-anchored NPs present in solution. This effectively reduced their net uptake into HepG2 cells. In contrast, no change was exhibited by Dox solution (12.74% ± 0.15) and NPs (25.95% ± 0.09), thereby confirming the ASGPR uptake was effected by the ligands Pul, AGn, and Pul-AGn combination. In an earlier study, the association of galactose terminal superparamagnetic iron oxide nanoparticles (SPIONs) to HepG2 cells was reduced by free galactose, supporting the model of ASGPR-mediated binding (Huang et al., 2008).

### Toxicity study

#### Body weight

Rats treated with Dox solution appeared weak, with hair erection, a hunched posture, and significantly (*p* < 0.05) low percent weight gain compared to the untreated control (Kalaria et al., 2009). In contrast, the nanoparticle-treated groups and untreated rats revealed comparable weight gain to untreated rats (*p* > 0.05), suggesting that Dox NPs were well tolerated by rats (Table 2). Furthermore, no change in weight was evident in the heart and kidneys in all the treatment groups.

**Table 2.** Percent body weight gain and weight of heart and kidneys of rats (mean ± SD; *n* = 5).

Treatment group	Weight gain% ± SD	Heartg ± SD	Kidneysg ± SD
Control	37.12 ± 4.88	0.90 ± 0.04	2.06 ± 0.10
Dox solution	9.13 ± 2.82	0.83 ± 0.02	2.04 ± 0.14
NPs	28.72 ± 5.02	0.88 ± 0.02	2.06 ± 0.09
AGn NPs	27.62 ± 6.23	0.88 ± 0.04	1.97 ± 0.09
Pul-AGn NPs	28.82	0.88 ± 0.04	1.98 ± 0.10

#### Evaluation of serum biochemical markers

The markers for renal (Supplementary Figure 1A) and hepatic (Supplementary Figure 1B) toxicity revealed no significant changes and were comparable to control in all the treatment groups (*p* > 0.05). CK and troponin I are considered primary markers of myocardial damage and hence cardiotoxicity.

Troponin I levels were comparable in all the groups (Supplementary Figure 1C, *p* > 0.05). On the other hand, significant enhancement seen in CK levels (*p* < 0.05) indicated cardiotoxicity of Dox solution group (Bertinchant et al., 2003; Xin et al., 2007), whereas comparable CK concentrations were seen in the control and nanoparticle groups (*p* > 0.05) confirming cardiosafety of the nanoparticles.

#### Histopathology of rat organs

Histopathological study ((Supplementary Figure 2) revealed moderate degree diffuse glycogen infiltration in liver, severe hemorrhagic spleen, and kidney with moderate to marked degree cloudy swelling of cortical tubules, marked reduction in glomerular filtration space in the Dox solution treated group. The nanoparticles and Pul NPs revealed no abnormality in the organs and were comparable to the control group suggesting safety. The AGn NPs showed moderate protein casts deposition in tubular lumen and tubular degeneration which was significantly lower in Pul-AGn NPs, suggesting possibility of mild renal toxicity with AGn.

### Efficacy study in PLC/PRF/5 liver tumor-bearing Nod/Scid mice

Mouse models of human cancer are increasingly employed for preclinical evaluation of anticancer efficacy. Subcutaneous xenograft models, which encompass implantation of human tumor cell lines or inoculation of biopsy material into the flank of immunodeficient mice or rats, have specific advantages of easy handling, high throughput, reproducibility, and are less time-consuming (Wartha et al., 2014). In the present study, PLC/PRF/5 liver tumor-bearing Nod/Scid mice were employed as a preclinical liver xenograft subcutaneous model of HCC. The PLC/PRF/5 cell line comes from a patient with primary liver cancer (Kato et al., 2005). The biological features of liver tumors induced by the PLC/PRF/5 cell line are reported to resemble hepatocellular carcinoma in humans very closely. Moreover, they exhibit high tumorigenicity (Sattler et al., 1982). Nod/Scid mice are combined T cell, B cell, and NK cell deficient. They also lack mature lymphocytes, serum immunoglobulin, MHC class 1 expression and hence tumors are easier to establish with faster tumor formation rate (Yan et al., 2013; Wartha et al., 2014).

#### Inhibition of tumor growth

**Figure 2.**
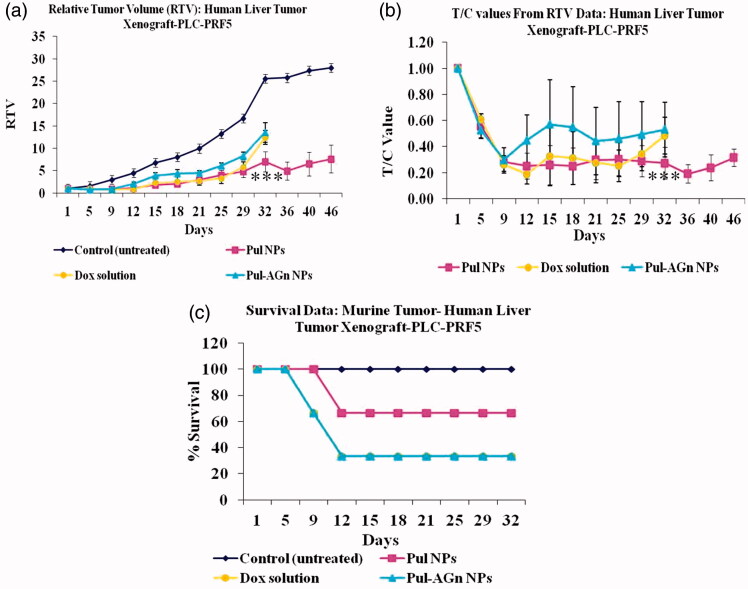
The inhibition effects of Dox formulations on the in vivo tumor growth. (a) The tumor growth curves for liver tumor-bearing nude mice after intravenous injections of Dox formulations, (b) T/C values, (c) survival plot (***p < 0.001).

All the treatment groups revealed suppression in tumor growth up to 12 days, whereas the untreated control group exhibited rapid and continuous increase in tumor volume (*p* < 0.001, k = 0.04 cm^3^/day) up to 46 days (Figure 2a). Comparable and slower regression was exhibited by Dox solution (k = 0.0078 cm^3^/day) and Pul NPs (k = 0.0061 cm^3^/day) up to 29 days. Nevertheless, beyond day 29, Dox solution exhibited rapid regression with relative tumor volume comparable to Pul-AGn group, whereas Pul NPs revealed sustained tumor volume up to 46 days indicating greater efficacy. In contrast, the Pul-AGn group revealed early regression (k = 0.0124 cm^3^/day) which was highly surprising considering the significantly enhanced hepatocyte uptake *in vivo* and *in vitro* uptake in HepG2 cell line which was nearly 2-fold than that seen with Pul NPs. In an earlier study with curcumin nanoparticles, we have shown rapid and early uptake followed by rapid elimination of AGn curcumin NPs in liver (D’Souza & Devarajan, 2016). The reduced efficacy of Pul-AGn NPs could therefore be due to early uptake in the liver, limiting the concentration in the tumor and hence limiting efficacy. Such distribution of a Pul-cisplatin prodrug (Wang et al., 2015) and Pul-based nanoparticles of methotrexate and combretastatin A4 (Wang et al., 2013) in both liver and tumor is reported in subcutaneous liver tumor-bearing nude mice. On the other hand, the stealth property of Pul coupled with (Guhagarkar et al., 2010; Wang et al., 2015) slower liver uptake (D’Souza & Devarajan, 2016) could have contributed to higher tumor accumulation and hence better efficacy.

The T/C ratio, a ratio of tumor volume in the treatment group (T) to the tumor volume in control untreated group (C), is an important indicator of antitumor efficacy, with T/C value ≤0.42 considered efficacious (LoRusso et al., 1990). It is seen from Figure 2(b) that value of <0.42 was evident for Pul NPs at all time points suggesting good efficacy. In contrast, T/C value exhibited by Pul-AGn NPs far exceeded 0.42 at most time points suggesting poor efficacy. Dox solution exhibited T/C values <0.42 up to day 29 with a sudden increase on day 32 to 0.48.

**Figure 3.**
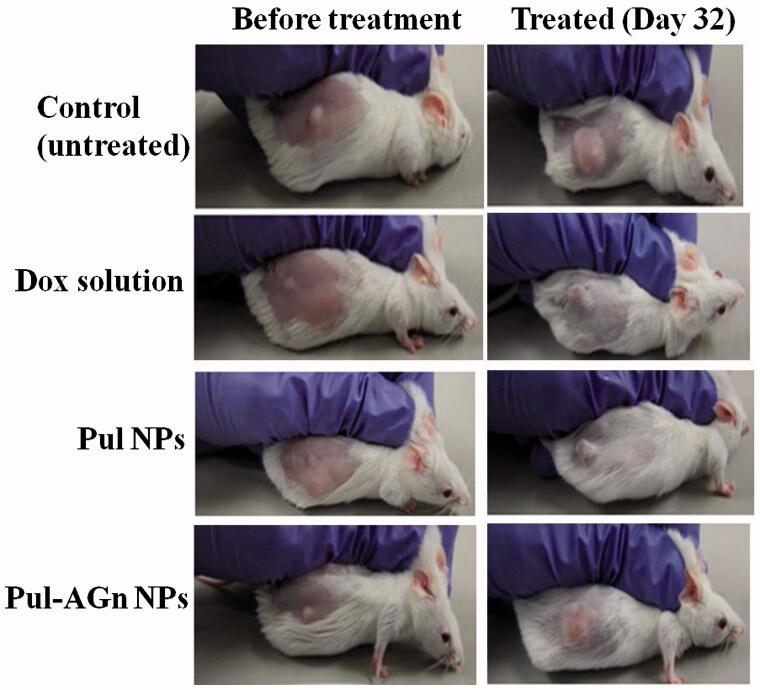
The picture of mice with tumor.

The superior anticancer efficacy with Pul NPs could also be attributed to affinity of Pul to integrin receptors and its effect on collagen (Pranatharthiharan et al., 2016). Figure 3 depicts the mice in the various treatment groups. Collagen is secreted in large quantities in tumor extracellular matrix, resulting in a stiff matrix which could readily exclude particles of size  >100 nm. Reduction of collagen in the HCC extracellular matrix (ECM) enabled improved penetration of particles into tumor matrix (Zhang et al., 2016). Local unfolding of the collagen triple helix is a primary requirement for cleavage of collagen (Manka et al., 2012). Changes in collagen structure or its cleavage can lead to decreased interstitial fluid pressure and hence improved intratumoral penetration of nanoparticles (Sagnella et al., 2014). Furthermore, protein response through collagen unfolding could result in endoplasmic reticulum stress-mediated apoptosis (Vincenz et al., 2013). Poly(lactic-co-glycolic acid) (PLGA) NPs of cisplatin and rapamycin diminished collagen content in the tumor stroma of A375-luc melanoma xenograft tumor model leading to better perfusion of NPs with a notable antitumor efficacy (Guo et al., 2014).

**Figure 4.**
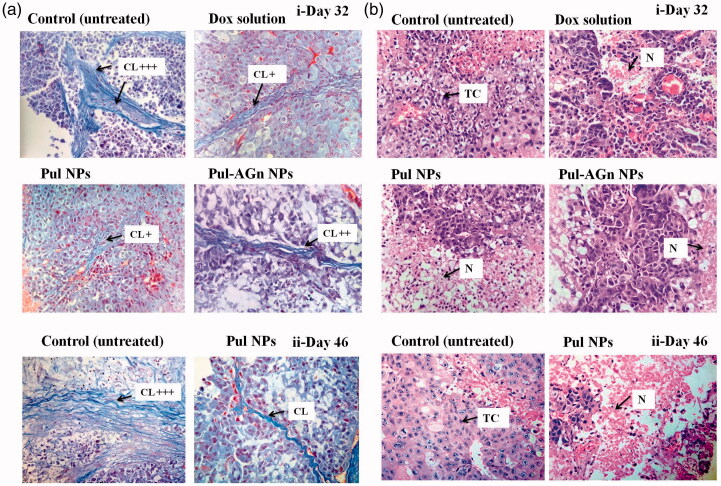
(a) Tumor sections were stained with Masson’s trichrome on day 32(i) and day 46 (ii) post first dose – the blue color represents collagen content. Original magnifications 400×. The collagen content was quantified using ImageJ and normalized to control (CL – collagen), (b) Images of H&E-stained tumor, sections excised from liver tumor-bearing mice following treatment with Dox formulations on day 32 (i) and day 46 (ii) post first dose. Original magnifications 400 × (TC – tumor cells with large prominent nuclei; N – necrotic cell mass).

In an earlier study, we demonstrated the pronounced efficacy of Pul NPs in the fibrosarcoma mouse model and attributed this to the effect of Pul on collagen in the tumor matrix (Pranatharthiharan et al., 2016). Reduced collagen density due to its manipulation by modification or reorganization is proposed as a promising strategy for anticancer efficacy (Eisinger-Mathason et al., 2013). Masson’s trichrome stain (Figure 4a) revealed high collagen content in the tumor of untreated (control) group (collagen normalized to control, day 32: 1 ± 0.2, day 46: 1.02 ± 0.33), whereas the collagen content was comparable (*p* > 0.05) and significantly lower (*p* < 0.05) in all the treatment groups (day 32, Dox solution: 0.51 ± 0.03, Pul NPs: 0.42 ± 0.09, Pul-AGn NPs: 0.49 ± 0.06; day 46, Pul NPs: 0.27 ± 0.22).

Yet another advantage of Pul NPs was the lower mortality seen with Pul NPs compared to the other groups (Figure 2c). Histopathology of tumor revealed that untreated control group (Figure 4b) showed large number of cancer cells with highly invasive tumor growth. The tumor region comprised of viable tumor cells that were low differentiated with high nuclear–cytoplasmic ratio, and presence of abundant mitotic figures indicating successful establishment of tumor. Following treatment, on day 32, a remarkable rise in necrotic area in multiple regions of tumor with Pul NPs compared to other groups indicated better interstitial diffusion within the tumor (Figure 4bi) confirming its superior efficacy. Similar enhancement in necrotic region indicating broad interstitial diffusion within the tumor was reported earlier with cationic SMEDDS of etoposide (Shete et al., 2015). Necrotic regions were low with Dox solution and marginal with Pul-AGn NPs. Furthermore, the necrotic region increased significantly in the Pul NPs group on day 46 (not monitored in other groups due to mortality) confirming sustained efficacy with Pul NPs (Figure 4bii). However, tumor vasculature improved in all the treatment groups compared to the untreated control group (Torosean et al., 2013; Neijzen et al., 2015).

Mitotic index has been proposed as an auxiliary prognostic factor in patients with HCC as high mitotic index correlates with larger tumor size (Ha et al., 2016). The average mitotic index % for the untreated control of 5.708 on day 32 increased significantly to 7.3 on day 46. All the treatment groups on day 32 revealed comparable mitotic index (Dox solution: 3.338, Pul NPs: 3.066, Pul-AGn NPs: 3.174). However, on day 46, Pul NPs revealed a significant reduction to 1.135 indicating significant inhibition in tumor growth (Alexander-Bryant et al., 2015).

#### Evaluation of serum α-fetoprotein

Serum α-fetoprotein (AFP) is a primary serum biomarker for HCC and more than 90% of the patients with hepatic cancer have increased serum AFP levels. It is a 72 KDa α-1 globulin and measurable only in minuscule amounts in the serum of normal adults (Zinkin et al., 2008). Serum AFP levels mirror tumor response to HCC chemotherapy and reduced levels of this marker correlate with decrease in the rate of tumor development (Langeswaran et al., 2013). Survival benefit has been associated with fall in AFP levels (Bialecki & Di Bisceglie, 2005). Normal mice exhibited negligible levels of α-fetoprotein in serum (<1 ng/ml). The untreated control showed marked increase in serum α-fetoprotein levels (Supplementary Figure 3, *p* < 0.01). Although Dox solution revealed significantly lower levels of AFP (*p* < 0.05) compared to untreated control, the ligand-anchored nanoparticles, Pul NPs, and Pul-AGn NPs exhibited levels that were significantly lower than Dox solution (*p* < 0.05). Furthermore, no significant increase in AFP level was seen even on day 46 with Pul NPs (*p* > 0.05). The remarkable reduction in serum AFP levels with Pul NPs in conjunction with improved survival compared to other treatment groups reflects its high efficacy.

#### Side effect analysis

Reduction of side effects of drugs is an important goal of targeted drug delivery. Body weight changes in mice are associated with systemic toxicity and the variation in body weight of mice of all groups is shown in Supplementary Figure 4. A significant reduction in weight of almost 30% (*p* < 0.01) compared to all other groups was seen with Dox solution indicating systemic toxicity. The untreated control revealed low weight loss which could be attributed to increased tumor weight. Weight loss exhibited by Pul NPs and Pul-AGn NPs was significantly lower and comparable (*p* > 0.05) with a maximum weight loss of 10% in the initial period of about 9 days followed by weight gain. Sustained release of Dox from nanoparticles prevented the overexposure of drug to the normal cells, thereby decreasing its toxicity. Similar observations of no body weight changes in nanoparticle group are attributed to sustained release of drug from nanocarriers as reported earlier (Long et al., 2014).

Nephrotoxicity and cardiotoxicity are two major adverse effects of Dox chemotherapy. As shown in Supplementary Figure 5i on day 32, Dox solution group revealed tubular necrosis, vascular congestion, and reduction in Bowman’s space with severe histopathological alterations. The untreated control group showed moderate tubular degeneration, whereas Pul-AGn NPs showed moderate reduction in Bowman’s space. In contrast, Pul NPs showed normal glomeruli and tubules comparable to normal mice with no evidence of toxicity. On day 46 (Supplementary Figure 5ii), although untreated control group revealed tubular degeneration, significant reduction in Bowman’s space, and tubular swelling, Pul NPs were found to be safe. The heart tissue slice of the Dox solution group on day 32 showed vascular congestion, degeneration of cardiac muscle, and infiltration of inflammatory cells with severe histopathological changes, whereas Pul-AGn NPs revealed mild infiltration of inflammatory cells. Pul NPs exhibited mild vascular congestion, nevertheless, histopathology showed normal cardiac muscles and cardiac endometrial endothelium comparable to normal mice suggesting adequate safety. On day 46, both control untreated group and Pul NPs group revealed normal cardiac muscles and cardiac endometrial endothelium.

## Conclusion

We present Pul Dox NPs as nanocarriers for ASGPR-mediated targeted therapy of HCC. The high efficacy coupled with the low toxicity presents great promise of this system.

## Supplementary Material

Supplementary_figure_5.tiff

Supplementary_figure_4_Changes_in_body_weight.tif

Supplementary_figure_3_Serum_AFP.tif

Supplementary_figure_2_Histopathology_rats.tiff

Supplementary_figure_1_Serum_Biomarkers.tif

## Notes

**Supplementary material available online**
