# Supplementary figures and images for: Asialoglycoprotein receptor targeted delivery of doxorubicin nanoparticles for hepatocellular carcinoma
Source: Drug Deliv. 2017 Feb 3;24(1):20–9. doi: 10.1080/10717544.2016.1225856 (PMC8244555; doi:10.1080/10717544.2016.1225856)

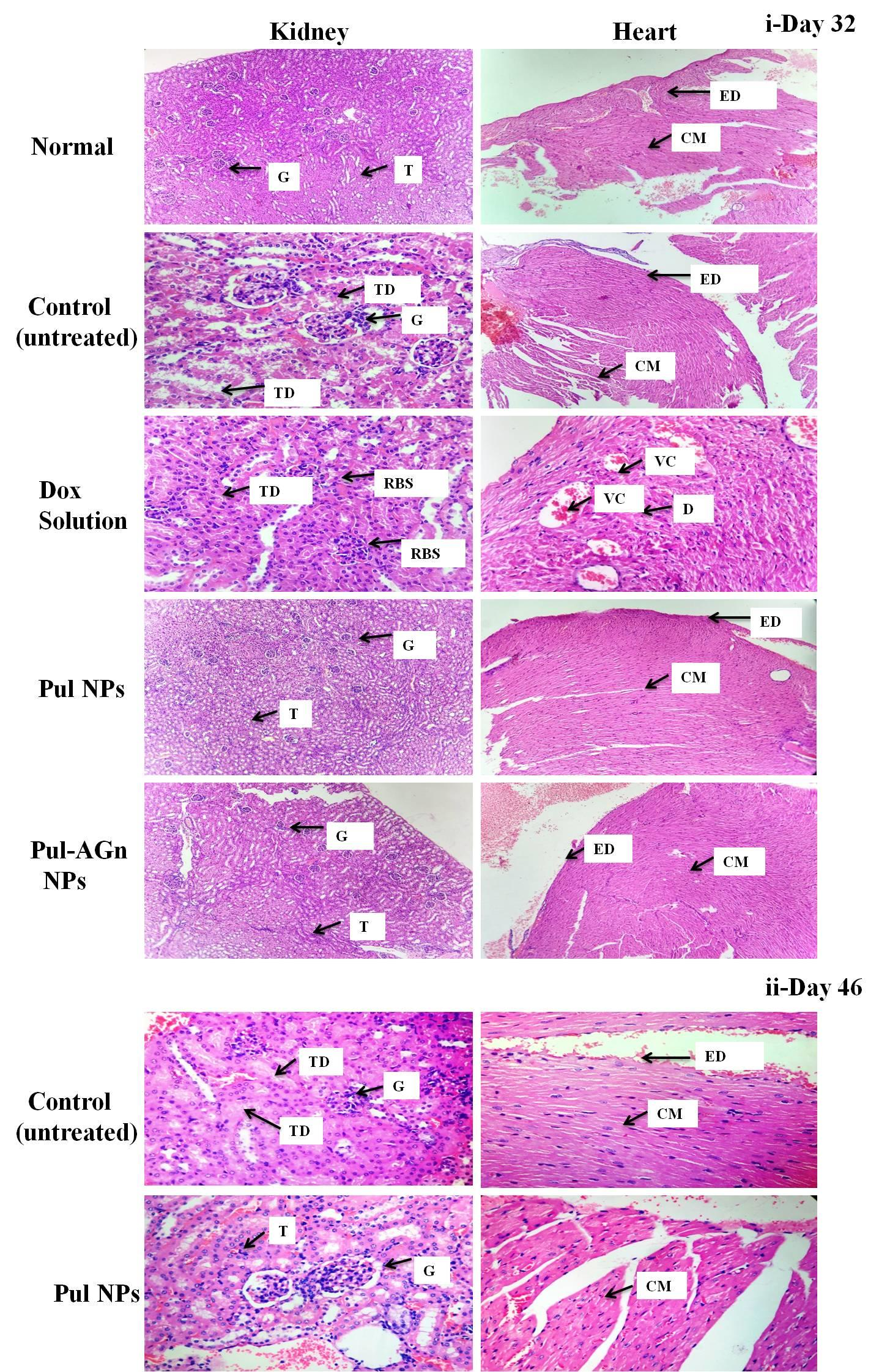

Supplement: Supplementary_figure_5.tiff [file IDRD_A_1225856_SM5198.tiff]

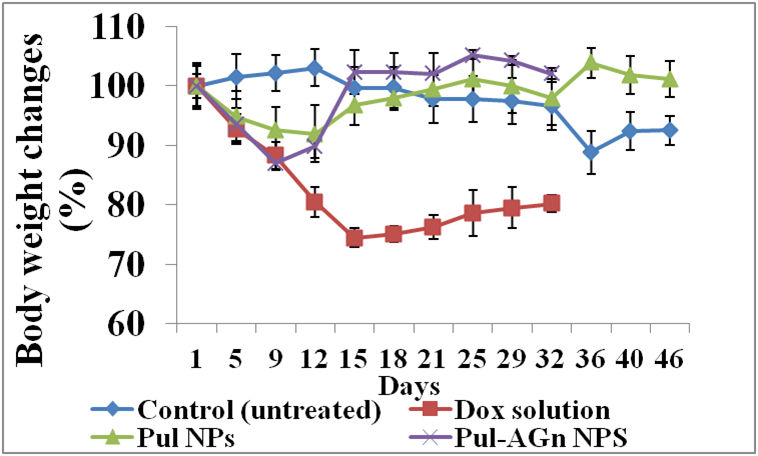

Supplement: Supplementary_figure_4_Changes_in_body_weight.tif [file IDRD_A_1225856_SM5195.tif]

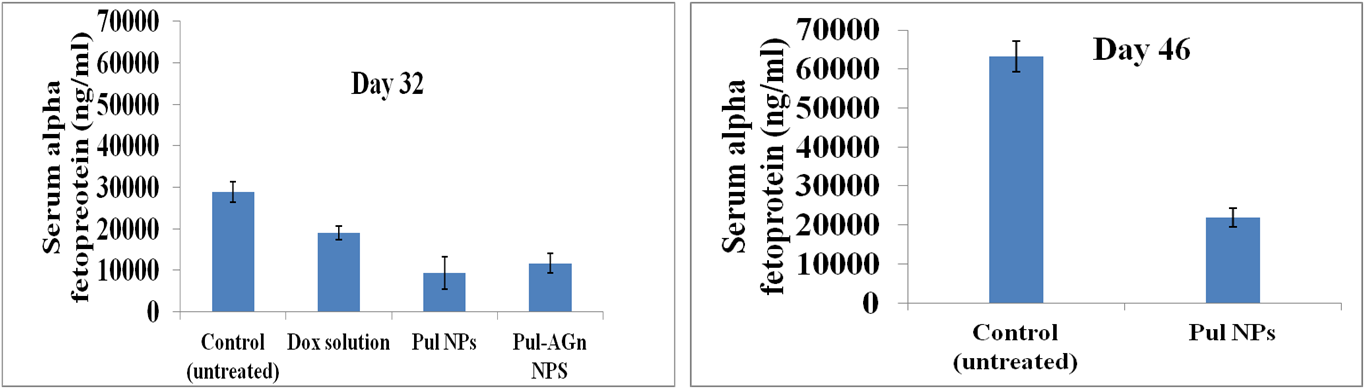

Supplement: Supplementary_figure_3_Serum_AFP.tif [file IDRD_A_1225856_SM5194.tif]

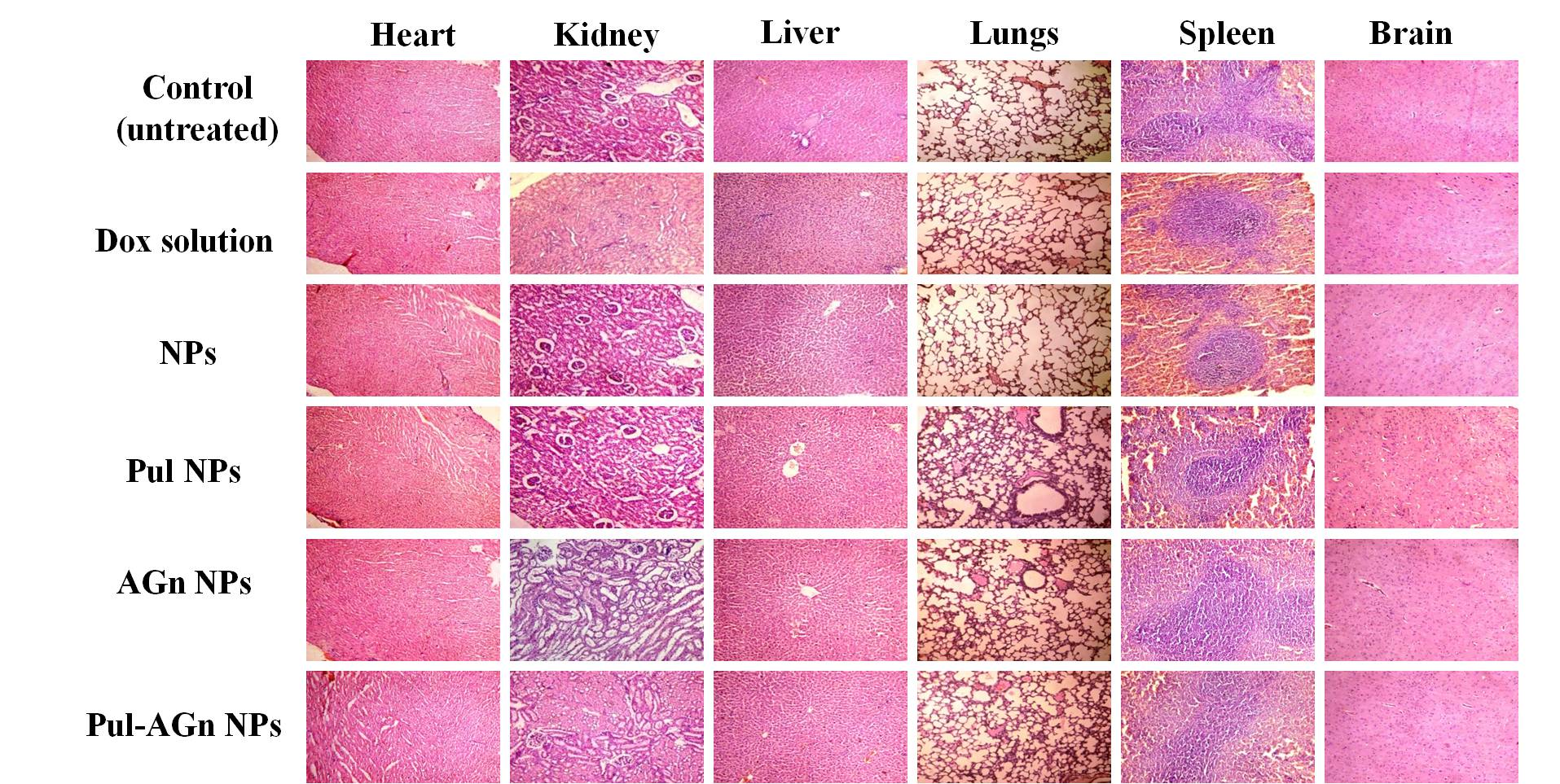

Supplement: Supplementary_figure_2_Histopathology_rats.tiff [file IDRD_A_1225856_SM5191.tiff]

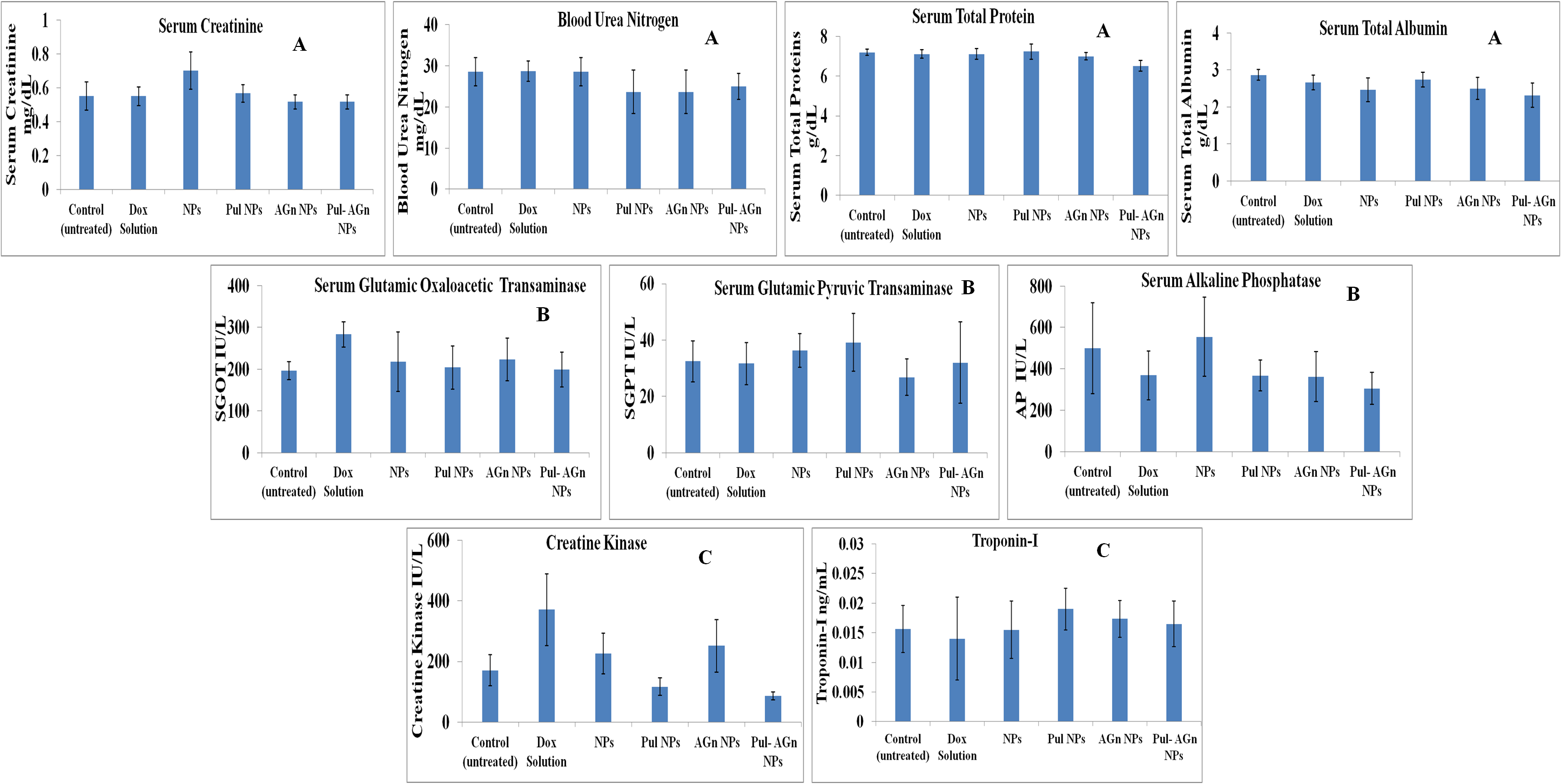

Supplement: Supplementary_figure_1_Serum_Biomarkers.tif [file IDRD_A_1225856_SM5185.tif]
